# Novel Monitoring Techniques for Characterizing Frictional Interfaces in the Laboratory

**DOI:** 10.3390/s150509791

**Published:** 2015-04-27

**Authors:** Paul A. Selvadurai, Steven D. Glaser

**Affiliations:** University of California, Berkeley, CA 94720, USA; E-Mail: glaser@berkeley.edu

**Keywords:** multicontact interface, pressure-sensitive film, normal stress distributions, finite element modeling, photometry, acousto-optical method

## Abstract

A pressure-sensitive film was used to characterize the asperity contacts along a polymethyl methacrylate (PMMA) interface in the laboratory. The film has structural health monitoring (SHM) applications for flanges and other precision fittings and train rail condition monitoring. To calibrate the film, simple spherical indentation tests were performed and validated against a finite element model (FEM) to compare normal stress profiles. Experimental measurements of the normal stress profiles were within −7.7% to 6.6% of the numerical calculations between 12 and 50 MPa asperity normal stress. The film also possessed the capability of quantifying surface roughness, an important parameter when examining wear and attrition in SHM applications. A high definition video camera supplied data for photometric analysis (*i.e.*, the measure of visible light) of asperities along the PMMA-PMMA interface in a direct shear configuration, taking advantage of the transparent nature of the sample material. Normal stress over individual asperities, calculated with the pressure-sensitive film, was compared to the light intensity transmitted through the interface. We found that the luminous intensity transmitted through individual asperities linearly increased 0.05643 ± 0.0012 candelas for an increase of 1 MPa in normal stress between normal stresses ranging from 23 to 33 MPa.

## 1. Introduction

Structural health monitoring (SHM) using non-destructive techniques (NDT) is a continually developing field of study with immediate and important benefits to the general population. The field is driven by balanced studies using experimental and theoretical aspects. In most cases, the field is driven by new sensors and sensing techniques, which allow us to develop a better understanding of the external forcing facing structures in either a continuous or iterative manner. In this study, we develop a novel method to better understand frictional processes and their degradative influences on a structure's health. A better understanding of friction and the means to assess (1) wear (surface to surface interactions); (2) attrition (erosion by friction) and (3) the proper assemblage of structural components (e.g., pressure vessels, pipe flanges, structural joints) could have long-term impacts on the structural health and efficacy of operations.

Friction from the contacts occurring between solids involves both the interaction of mechanical and geometric properties [1]. Accurate understanding of this problem has eluded physicist, engineers and scientists since the first well-known experiments conducted by Leonardo da Vinci (*ca.* 1500) [2]. While the simplistic versions of Amontons–Coulomb laws of friction were sufficient until the late 1950s, it was not until the following developments of Bowden and Tabor [3] and Rabinowicz [4], who discussed the importance of plasticity in contact, Archard [5] and Greenwood and Williamson [6], who characterized the surface roughness and interactions using statistical mechanics, that contacts along the multicontact interface (MCI) were shown to form in three mechanical constitutive behaviors: (I) elastic; (II) elasto-plastic; and (III) fully plastic [7]. For dry, unlubricated friction, the only manner for stress to be transmitted across the interface is via the junction regions that are dispersedly located along the fault, so the mesoscopic motions of the interface must be described by the interaction and movements of multiple asperities at the asperity-level.

In the late 1970s, Dieterich [8] and Ruina [9] developed the so-called rate and state constitutive laws (RS) to describe the manner in which mesoscopic motions are dictated by the numerous contacts formed along the MCI. Their field of study was rock mechanics, and the RS law was developed in the laboratory with the goal of understanding the earthquake cycle in a mechanical manner. Their model has been shown to capture the most prominent features of the seismic cycle, but its applicability is still widely debated [10,11]. The promising features of the RS model are that it is a unifying law, largely material-independent (applies for paper-paper, Lucite-Lucite, wood-wood and rock-rock interfaces among others [12]) and applicable down to the microscopic level. However, one of the goals and reasons for the continued study of dry frictional sliding at low-velocities in the laboratory is the search for mechanistic constitutive laws. Feedback and improvement are allowed by improvement in sensors and sensing techniques in the laboratory.

**Figure 1:**
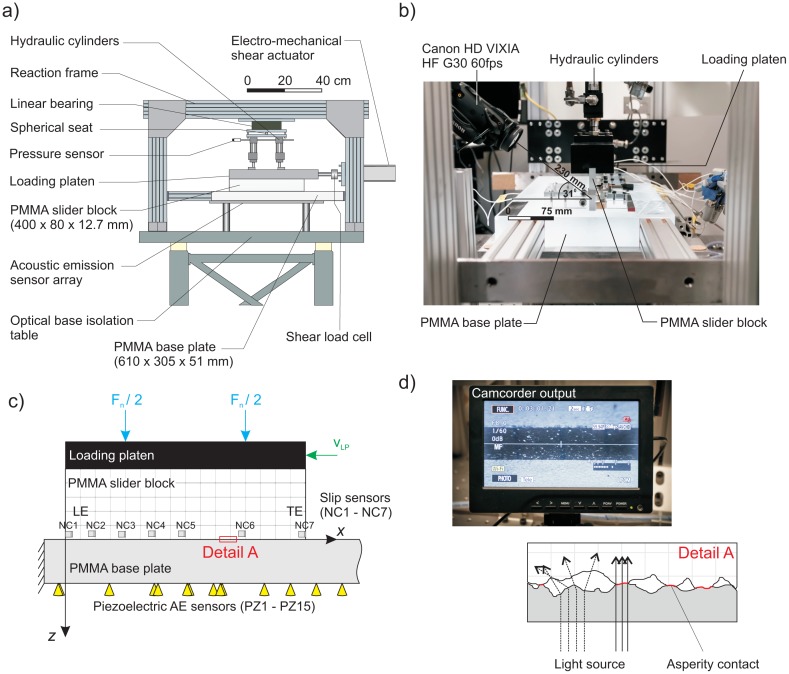
(**a**) An overall schematic representation of the direct shear friction apparatus and its major components; (**b**) a photograph of the apparatus showing the location of the video camera in relation to the frictional multicontact interface (MCI); (**c**) general configuration of the direct shear apparatus from the side view with general locations for the non-contact eddy current (NC1–NC7) and acoustic emission (AE) piezoelectric sensors (PZ1–PZ15). These sensors were used to detect dynamic changes as the fault began to fail; (**d**) (top) A photograph of the monitor used to display the video camera images in real time. Asperities appear as the bright spots within the darker, interface region. (bottom) A schematic representation of Detail A from (**c**) depicting the theory, which describes the transmission of light through asperities (solid lines) and diffracted light along the void space (dashed lines).

In this study, we present methods to improve our ability to understand how asperities are formed along the multicontact interface (MCI). Here, we develop unique techniques to characterize contact interactions, specifically their stress states. Our experiment consists of two PMMA samples, a slider and a base block (Figure 1a), which when pressed together, form an MCI due to the interaction of the rough surfaces (Detail A in Figure 1d). Two methodologies have been developed to understand the intricacies of asperity formation under simple normal loads. We first use a pressure-sensitive film to characterize the normal stresses on the contacting asperities. The pressure-sensitive film provides the detail of both the heterogeneity along singular asperities and the relative distances between larger asperities, which dominate the transfer of stress across the interface. Secondly, we use the transparent nature of the PMMA to our advantage. The interface could be visualized (Figure 1b) through a video camera focused on the interface through the side of the PMMA slider block. Dieterich and Kilgore [12–14] used a similar technique to image individual contacts on transparent PMMA samples. They observed that light was transmitted through the asperities, which appeared brighter in images (Figure 1d, top) than the non-contacting background. We use a similar technique, but focus on the subtle variations in transmitted light through the asperities, which is indicative of variations in the normal stress field at the contact junction (*i.e.*, the millimetric length scales).

We found that as higher normal stress is observed over singular asperities, a “better” bond is achieved, allowing for better transmission of light, which can be detected using the video camera. We could then compare the light transmitted through individual asperities whose normal stresses were also calculated beforehand using the pressure-sensitive film. This allows quantification of changes in transmitted light to changes in normal stress, an indicator of the asperities' shear strength in terms of contact mechanics [7]. This is important when trying to quantitatively understand the local variations in stress states causing individual asperities to fail dynamically when the interface is slowly sheared [15-17]. Results for the slow shearing of the interface are presented in the latter sections of this study.

We performed a full direct shear experiment with along-fault strain changes monitored using slip and the foreshocks released by asperity slip recorded by an array of Glaser-type acoustic emission sensors. A camera was focused on the region where sudden changes in asperity luminosity were corollary to the failure (or partial failure) of singular asperities. The failure of these asperities were located in space and time with respect to the video camera images, and the methodology used to capture this transition is discussed.

## 2. Experimental Facilities

### 2.1. General

In the experiments presented here, dry and unlubricated conditions along the fault were carefully controlled during each experiment. The tests consisted of loading two samples of PMMA in the direct shear apparatus detailed in Figure 1a. To create the frictional interface, a far-field normal force *F_n_* was applied via two hydraulic balanced cylinders (Parker H3LLT28A) to the PMMA slider block (400 mm × 80 mm × 12.7 mm) through a rigid loading platen. An in-line pressure transducer (OMEGADYNE PX329-2KG5V) was connected to the cylinders and was used to measure the pressure, then converted to a force. In the first part of the experiments conducted here, the interface was not sheared, and the rigid loading platen was held stationary (*V_LP_* = 0 mm/s) using the shear actuator (Exlar Tritex IIT2X115). During the shearing of the interface, discussed in the final part of this study, the shear actuator drove the rigid loading platen at *V_LP_* = 0.007 mm/s.

### 2.2. Instrumentation

The general arrangement of sensors used in this investigation is shown in Figure 1c. We used non-contact eddy current induction sensors (Shinkawa VS-020L-1) at seven locations along the fault (NC1-NC7) to measure the macroscopic longitudinal strains along the fault. Two eddy current sensors were placed at the leading (LE) and trailing edge (TE; the edge in contact with the thruster) of the slider block in the y-direction. These allowed us to index the slider block with respect to the base plate and measure for any twisting during failure of the interface. Aluminum targets were used to change the inductance of the magnetic field, which was linearized using a displacement converter (Shinkawa VC-202N), over frequency ranges from 0 to 20 kHz (−3 dB), and showed a linearity of ∼444.52 ± 0.11 μm/V between 0 and 2000 μm with a resolution of 0.1 μm for each sensor. Mounting of the targets to the sample was done with a minimal amount of cyanoacrylate in a similar manner to Ohnaka and Shen [18].

An array of 15 Glaser-type conical acoustic emission sensors (PZ1-PZ15) was placed on the underside of the base plate (Figure 1c). The detailed layout of the acoustic emission array and non-contact sensor array can be found in Selvadurai and Glaser [17]. These sensors were absolutely calibrated beforehand using the techniques of McLaskey and Glaser [19,20]. They detect surface normal displacements from a near-field vantage point with a frequency bandwidth of ∼5 kHz−2.5 MHz (−3 dB) with ±1 pm noise floor [19]. An ELSYS TraNET EPC 16-bit dynamic range, 40-MHz sample rate, high-speed digitizer measured the high-frequency stress waves emitted during the impulsive destruction of asperities.

### 2.3. Sample Preparation

The surfaces of the PMMA slider and base are machined flat (∼0.001” over 16”), then professionally sandblasted using 40–60 grit Al2O3 (440–220 μm) to create roughness profiles [21] similar to those found in nature [22]. Before any reported testing, the interface was sheared for a cumulative slip of ∼36.1 mm. This ensured the formation of a mature frictional interface; at this point, the changes from abrasive and adhesive wear [11] become minimal and do not control friction [23], which is associated with a mature faulting surface.

### 2.4. PMMA Properties

Polymethyl methacrylate is a glassy polymer [24] with physical characteristics at room temperature as follows: density ρ = 1180 kg/m^3^, shear modulus *G* = 1.8 GPa and a Poisson's ratio of ν = 0.3. The body and shear wave velocities are 2700 m/s and 1390 m/s, respectively. Using digital scanning calorimeter (DSC), we determined the glass transition to be *T_g_* = 114 °C. We determined all of the above values independently, and they fell within the range of values of PMMA material properties found in the literature [24,25].

### 2.5. Photography of Interfacial Asperities

The unique setup and transparent properties of the “glassy” PMMA allowed us to directly observe the interface during the experiments. Figure 1b depicts the location of the video camera (VIXIA HF G30 CMOS) in relation to the faulting plane. The video camera was focused at a 31° angle to the horizontal and a distance of 230 mm from the frictional plane. Detail A in Figure 1d illustrates the theory of operation [13]: (I) two nominally flat interfaces only touch asperities; (II) the light was transmitted more effectively through these contacting asperities; and (III) light was diffracted through the void space in which no contact occurred. The high definition video camera operated at a video frame rate of 60 fps (Δ*t_frame_* = 1/60 s ∼ 16.7 ms), and images were obtained at a focal length of 3.67 mm. To improve image resolution without the use of digital enhancement, a CANON 58 mm Close-up Lens 250D was attached on the camera directly. The field of view was 10 mm, and images are 2136 × 1362 pixels per frame, making the resolution 0.005 mm/pixel (∼5 μm/pixel). No image correction was performed for the optical distortion; instead 200 μm-thick lines were drawn along the interface at 5-mm spacing, forming a stereographic grid to account for the orthoscopic distortions from the camera, close-up optical lens attachment and light passing through the PMMA sample simultaneously.

### 2.6. Pressure-Sensitive Film

We employed a FUJI Prescale pressure-sensitive film to detect and measure the contacting asperities formed between the interacting surfaces. The film is polyethylene based and has a thickness *b* of approximately 90 μm. The film has embedded microcapsules (5-μm resolution) that, when compressed, release ink with colors proportional to the applied pressure (±1.5 Pa). The film is rated for normal pressures between 12 to 50 MPa according to the manufacturer. At stresses higher than 50 MPa, the microcapsules do not further discolor, and the intensity is saturated (*I* **∼** 0.3 candelas (cd)). After loading in the fault, the film was digitized using an image scanner and algorithms in MATLAB to detect, size and catalog all contacting asperities in the static state, under normal load and not subjected to shear. The film was calibrated using a finite element model (FEM) in the commercially available software ABAQUS, and the results are presented in Section 3.

## 3. Calibration of the Pressure-Sensitive Film

### 3.1. Indentation Test

**Figure 2:**
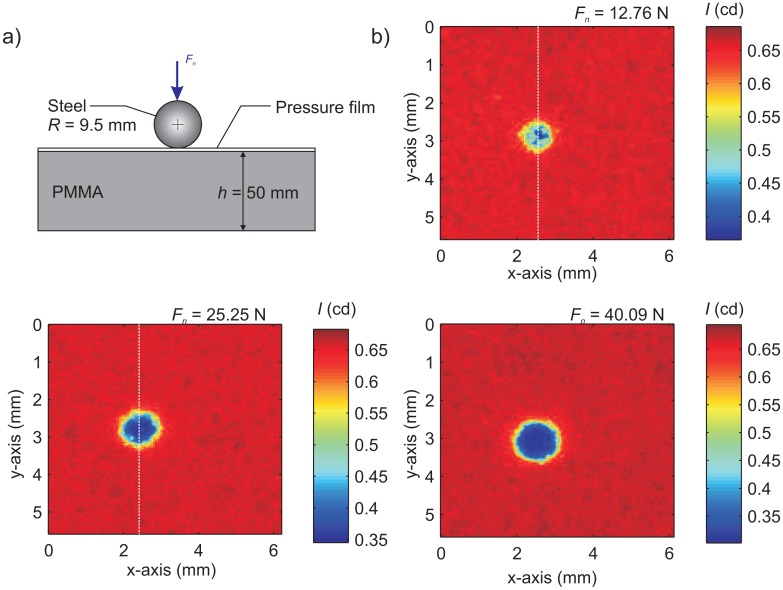
(**a**) Indentation test configuration used to calibrate the pressure-sensitive film; (**b**) digitized images of the pressure-sensitive film for various levels of normal force applied through the steel indenter. Digitized images were converted to luminous intensity and had an absolute range of 0–1 candelas (cd).

To test the pressure-sensitive film, we used the spherical indenter apparatus shown in Figure 2a. The pressure-sensitive film was used to measure the normal stress profiles induced from a spherical steel indenter (radius *R* = 9.5 mm), which compressed the film against a 50 mm-thick PMMA base plate. The substrate was assigned a Young's modulus *E* = 1.8 GPa [25] and a Poisson's ratio ν = 0.3 [24]. The apparatus was capable of delivering a normal force *F_n_* from 0–70 N (compression), which was measured using an in-line load cell (ELPF-T3M-500N). A new piece of pressure-sensitive film was used for each level on normal force, since once the film discolored, it no longer had the ability to measure lower normal stress levels. A small section of 5.5 mm × 6.1 mm surrounding the discolored contact patch resulting from contact was digitized using an HD scanner (MUSTEK SE A3 USB 2400 Pro) at 24-bit RGB color. Once digitized, the image was imported into MATLAB [26] and each pixel converted from an RGB color scheme to the luminous intensity color scheme [27]. Luminous intensity is an SI (International System) photometric measurement unit that has a value between 0 and 1 cd and refers to an average measurement of light intensity across the visible spectrum. This metric is known as photometry and was also employed for the video camera in the following sections. Figure 2b shows the luminous intensity for three indentation tests at *F_n_* ≈ 13, 25 and 40 N. The brighter region (outside the contact patch) has a higher value of *I* ∼ 0.65 cd, while the darker contact region varies from *I* ∼ 0.3–0.6 cd.

### 3.2. Numerical Modeling

**Figure 3:**
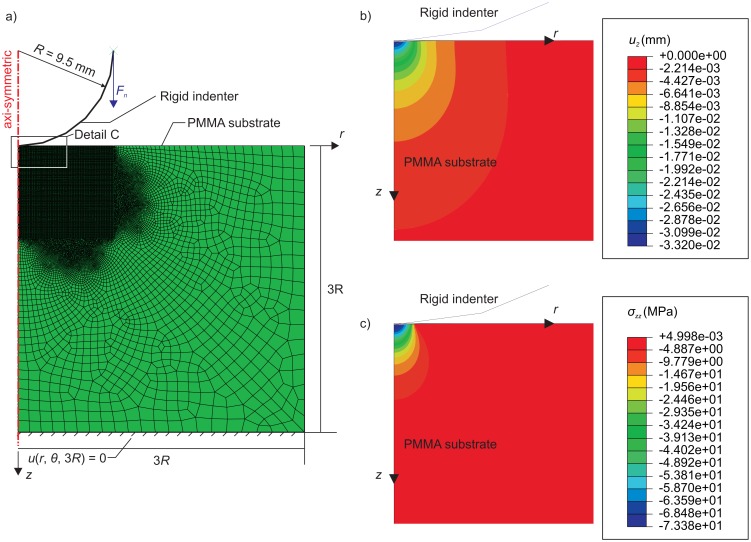
(**a**) Numerical model used to model the pressure distributions measured by the pressure-sensitive film; (**b**) displacements in the *z*-direction (*u_z_* = *u*_2_); and (**c**) normal stress (σ*_zz_*) from Detail C in (a). The results in (b) and (c) are for an applied normal force of 49.74 N.

To better understand the normal stress distributions seen in Figure 2b, an axisymmetric, 2D finite element model was created using the commercially available ABAQUS software. The model excluded the effects of the film and frictionless contact assumed between the rigid indenter and PMMA substrate. At room temperature (∼25 ^°^C), we do not expect the material to yield (or experience any softening) until ∼78 MPa [24], and a linear elastic model was used. The deformations were modeled as classical Hookean isotropic elastic [28]. Incremental elastic strains are given by,

(1) $$d\varepsilon_{ij}=\frac{d\sigma_{ij}}{2G}-\frac{\lambda^{*}\cdot d\sigma_{kk}\delta_{ij}}{2G\left(3\lambda^{*}+2G\right)}$$

where λ^*^ is the Lamé's first parameter and *G* is the shear modulus, and summation over the repeated indices is implied. The model is composed of 46,687 quad elements and was refined to a length scale of 100 μm in the region of contact; it is presented in Figure 3a, as are the boundary conditions. Detail C in Figure 3a is enhanced and shows the *z*-direction component of displacement (*u_z_* = *u*_2_) (Figure 3b) and the normal stress component (σ*_zz_*) (Figure 3c) for an applied normal force *F_n_* = 49.74 N.

### 3.3. Numerical Modeling vs. Experimental Observations

**Figure 4:**
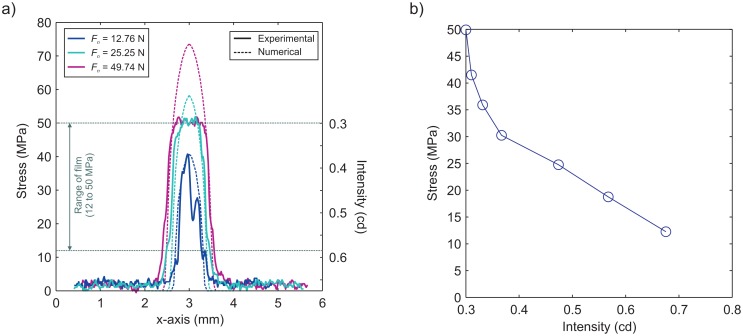
(**a**) Comparison of normal stress distributions from experimental pressure-sensitive film (solid lines) and the numerical model shown in Figure 3 (dashed lines) for various normal loads; (**b**) the relation between normal stress calculated numerically and the light intensity measured experimentally.

The distribution of pressures calculated by the model is shown in Figure 4a (dashed lines) for various normal loads for comparison with the measured one (solid lines). Transects were drawn through the centroid of contacts from the experimental pressures and compared with image intensity (Figure 4b). The centroid of contact was determined using the image detection algorithm in MATLAB. We see that the film saturates at 50 MPa, as was expected from the manufacturer's specifications. We calculated the errors between the experimental measurements and numerical results of the normal stress profiles at 12 MPa and 50 MPa and took the average error across the centroid in both the *x*- and *y*-directions for all three applied normal forces seen in Figure 4a. The maximum percentage error in the normal stress profiles ranged from −5%–6.6% at 12 MPa and ranged from −7.7%–0% at 50 MPa.

**Figure 5:**
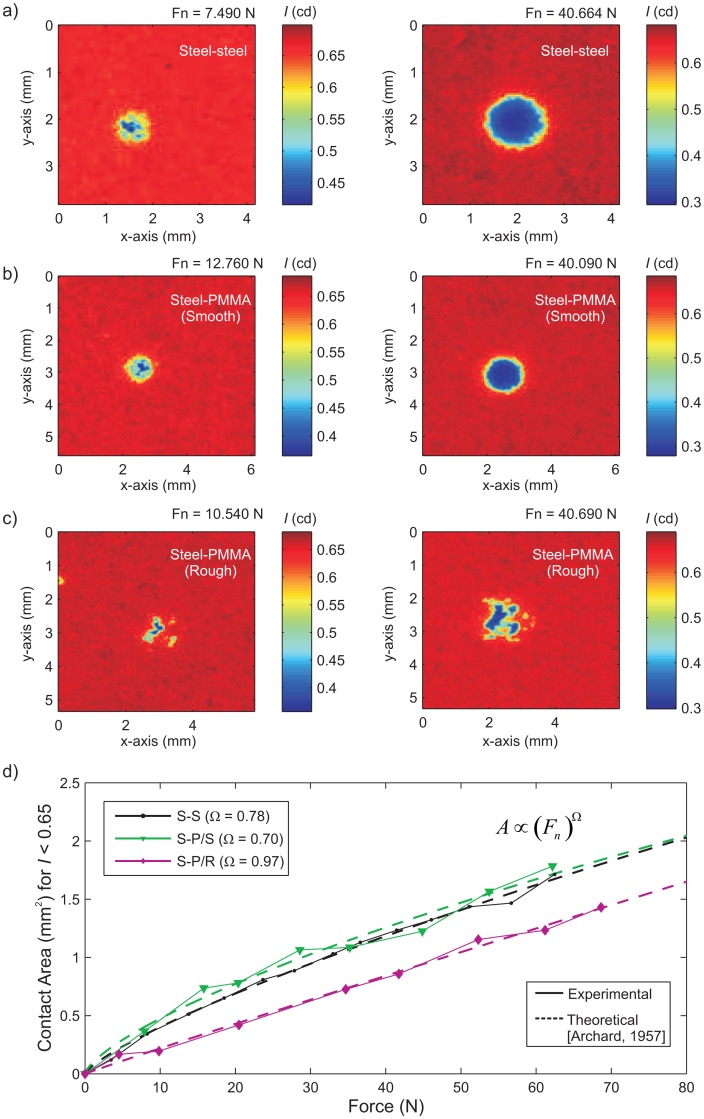
Light intensity (cd) obtained from the pressure-sensitive film for variable substrates pressed against with the same steel indenter. For a lower (∼10 N) and higher (∼40 N) applied normal force, we see the variations due to the: (a) steel substrate (S-S); (**b**) the smooth PMMA substrate (S-P/S); and (**c**) the rough PMMA substrate (S-P/R); (**d**) the comparison of the area, defined by pixels below the intensity threshold *I* = 0.65 cd, versus the applied normal force *F_n_*. The results are compared to the theoretical estimates made by Archard [5].

We suspect that some error in stress profile measurements may be induced by the fact that both the PMMA substrate and the steel indenter have roughness profiles at some length scale. Figure 5 shows images of normal stress distributions for indentation tests conducted at two normal load levels. Three different substrates were used in combination with the same steel indenter: (I) a polished steel substrate (S-S; Figure 5a); (II) a smooth PMMA substrate (S-P/S; Figure 5b); and (III) a rough PMMA substrate (S-P/R; Figure 5c). The rough PMMA substrate was sandblasted using 40–60 grit Al_2_O_3_ (440–220 µm), which had a similar preparation to the experimental interface used in the direct shear configuration shown in Figure 1a. Figure 5d shows the summation of pixels below light intensities of *I* < 0.65 cd at a wider range of applied normal force *F_n_*. The contact area was calculated knowing that each pixel had an area of 25 µm^2^ (5 µm × 5 µm). Archard [5] examined the effect of surface roughness on the elastic contact formed along an interface and found that the relation of the real contact area *A_r_* varied proportional to the applied normal force *F_n_*, and the relation is given as:

(2) $$A\propto {\left(F_{n}\right)}^{\Omega }$$

where Ω ranges from 2/3 to 1 for smoother to rougher surfaces, respectively. Figure 5d shows that Archard's [5] theoretical relation was comparable to the experimental measurements taken from the pressure-sensitive film. The steel-steel interface has Ω = 0.78 (*R*^2^ = 0.9962); the steel-PMMA/smooth interface has Ω = 0.70 (*R*^2^ = 0.9949); and the steel-PMMA/rough interface has Ω = 0.9721 (*R*^2^ = 0.9834).

## 4. Interfacial Measurements of Transmitted Light Using Photometric Methods

**Figure 6:**
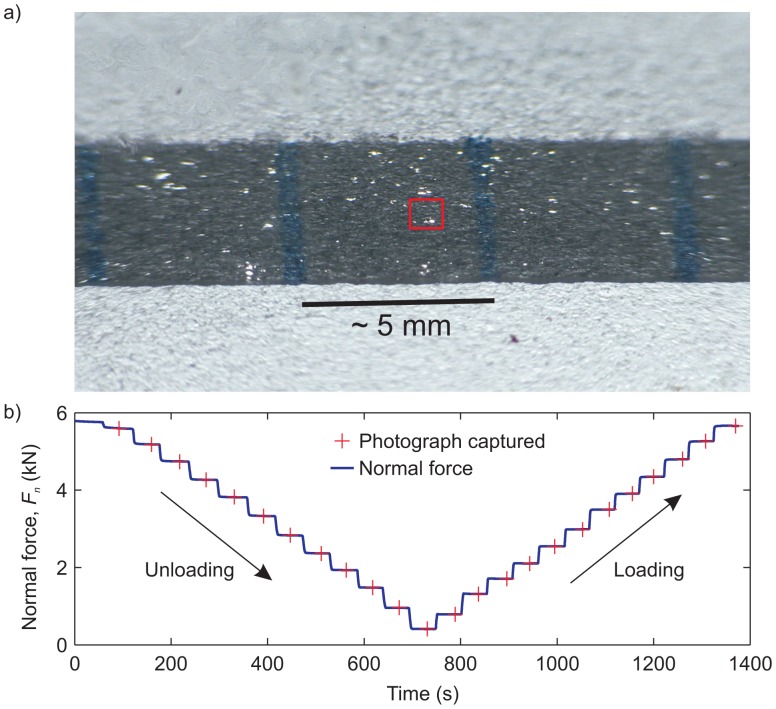
(**a**) An unprocessed image (2136 × 1362 pixels) from the video camera focused on the interface (darker region). Within the dark region are small bright regions that are asperities that transmit light more easily; (**b**) Test performed to measure the changes in light intensity and its relation to the applied normal force *F_n_*.

As mentioned in Section 2.5, we used a video camera to monitor the light transmitted through contacting asperities in the direct shear experimental configuration. In Figure 6a, we show an unprocessed image taken along the interface. The interface appears darker, and within the darkened section appear small populations of bright asperities. The edges of the image are distorted due to the lens' aperture effect. We focused our analysis on the portion of the image in the red box highlighted in Figure 6a.

### 4.1. Variations in Normal Load and Its Effect on Local Luminous Intensity

A test was performed to analyze the effect of light transmitted through asperities due to an increased far-field normal force *F_n_* in the direct shear setup shown in Figure 1a. The normal force was varied incrementally in a step-like manner, as seen in Figure 5b. At each level of normal force, a 2-s video (*i.e.*, 120 picture frames) was captured (red crosses in Figure 5b). The hypothesis is that as the far-field normal force increases, the normal stress at the asperity junction level increases. The normal force was first unloaded from *F_n_* ∼ 5.8 kN to 0.4 kN and then reloaded back to *F_n_* ∼ 5.8 kN. A total of 12 loading steps were performed for both the loading and unloading portions of this experiment. For each video, the central frame was used and processed to examine the changes in light intensity *versus* normal force. The unprocessed images captured from the camera (RGB format) were converted to luminous intensity (*I*) in MATLAB. An identical algorithm was used as for the pressure-sensitive film discussed in Section 3. To detect and catalog individual asperities, a lower threshold was set (*I_lower_* = 0.5 cd) for the same image detection algorithms applied to the pressure-sensitive film.

### 4.2. Results for the Normal Force Unloading-Loading Test

We focused on the small section of the interface shown in the red box of Figure 6a. The threshold for light intensity was set to *I_thresh_* = 0.5 cd. Examples of the images used are shown in the inset of the graph, and arrows indicate which data point they are associated with. We first examine the number of pixels above the threshold versus the applied normal force *F_n_*. The absolute number of pixels is not an indication of area, since the images are taken at an angle of ∼310°. However, we expect that the sum of light intensity above *I_thresh_* is related to the real contact area. If we assume that asperities form in a circular manner (e.g., Figure 5), the area will be proportional to the radius in the x-direction, and increasing the far-field normal force *F_n_* should cause asperities to grow [13], in turn leading to an increase in pixels transmitting light above *I_thresh_*. The added size of the asperity must balance the increase in *F_n_* for elastic, elasto-plastic or fully plastic asperity growth [7].

**Figure 7:**
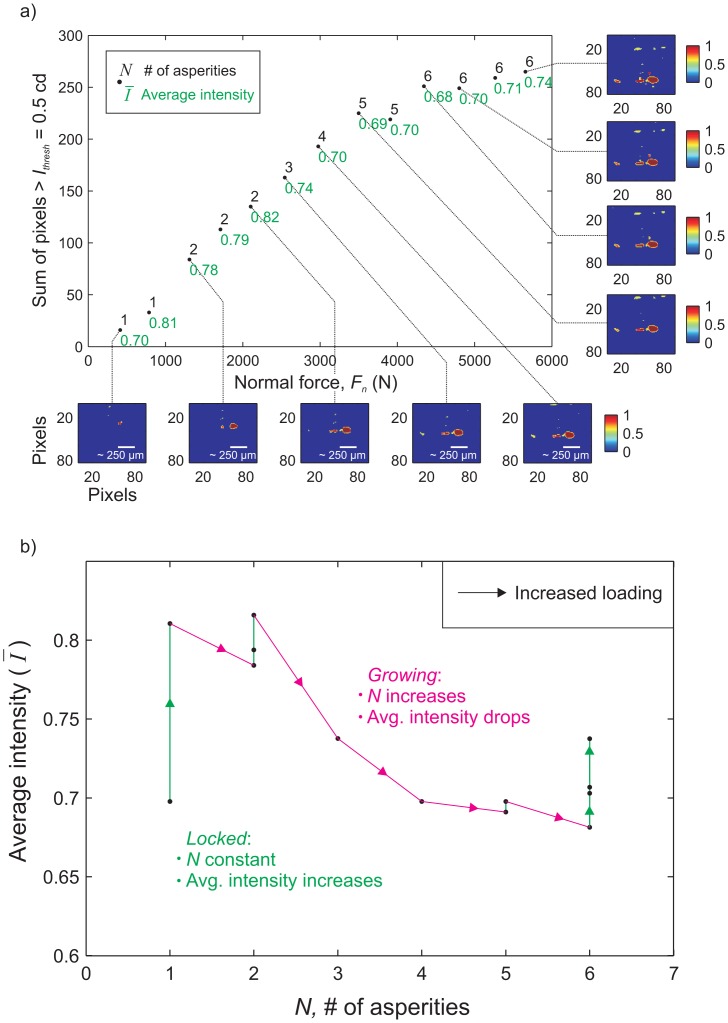
(**a**) Total pixels above the threshold light intensity *I_thresh_* versus the applied far-field normal force *F_n_*. The images along the axes are the processed images from the red box displayed in Figure 6a at various normal loads. For each loading step, the number of asperities *N* (black value) were calculated, using the image detection algorithm, and the average light intensity *Ī* over those asperities are given in green. (**b**) The average luminous intensity transmitted through the population of *N* asperities versus the number of total asperities in the population.

In Figure 7a, we show how a small population of asperities form in relation to increasing the normal force shown in Figure 6b. Within the highlighted red box (see Figure 6a), the image processing algorithm (see MATLAB function *regionprops* [27]) allowed us to measure: (I) the number of regions (*i.e.*, the number of asperities); (II) the number of pixels in each individual region; and (III) the distribution of light intensity in each region. To eliminate any background noise, a region (asperity) was defined as having a minimum of 10 pixels, above the intensity threshold *I_thresh_*, in direct contact with each other. The inset images in Figure 7a are snapshots of the highlighted region for respective levels of applied normal force *F_n_*. The study of asperities using their transmitted light intensity and our ability to catalog them individually using post-processing software has many benefits and can provide a number of interesting metrics to better understand the random process models of rough surfaces [10,29,30].

Since the seminal paper by Greenwood and Williamson [6], the study of asperity formation between randomly rough surfaces (GW model) have been extensively employed in numerous engineering and scientific endeavors. However, Greenwood has recently come forward [31,32], criticizing some of the assumptions made in this model and recent works. Primarily, the field of tribology is attempting to better understand these key criticisms. One critical assumption of the GW model is that asperities form independent of each other. The spacing between larger asperities is great enough that they do not interact as the normal force *F_n_* is increased. While interaction was believed to happen only when higher normal loads were applied, in truth, the process of asperity interaction is seen as prevalent. Studying the additional stresses introduced from the elastic interactions of asperities through the substrate are beginning to become important [10]. Newer studies on this subject are primarily computational interpretations [33–37], and experimental studies [38] are at the early stages of development.

Since the seminal paper by Greenwood and Williamson [6], the study of asperity formation between randomly rough surfaces (GW model) have been extensively employed in numerous engineering and scientific endeavors. However, Greenwood has recently come forward [31,32], criticizing some of the assumptions made in this model and recent works. Primarily, the field of tribology is attempting to better understand these key criticisms. One critical assumption of the GW model is that asperities form independent of each other. The spacing between larger asperities is great enough that they do not interact as the normal force *F_n_* is increased. While interaction was believed to happen only when higher normal loads were applied, in truth, the process of asperity interaction is seen as prevalent. Studying the additional stresses introduced from the elastic interactions of asperities through the substrate are beginning to become important [10]. Newer studies on this subject are primarily computational interpretations [33–37], and experimental studies [38] are at the early stages of development.

## 5. Combining Pressure Film and Photometric Measurements

We use the now calibrated pressure-sensitive film (Section 3) to examine the relation between luminous intensity across an asperity junction using the photometric methods (Section 4). Firstly, we employed the pressure-sensitive film in the direct shear configuration shown in Figure 1a. The fault is positioned at a datum location between the slider and base plate sample using the eddy current sensor array and fine-threaded screws [39]. Prior to applying a far-field normal force *F_n_*, a new strip of pressure-sensitive film was placed in between the two interface surfaces. The fault was loaded using a known applied far-field force *F_n_*, causing the pressure-sensitive film to develop. The pressure-sensitive film was developed for *t_hold_* = 900 s, and then, the fault was unloaded and the film carefully extracted and digitized. The calibration used in Section 3 was applied to determine the normal stress field along the fault. The frictional fault was carefully re-indexed to the same location using the eddy current sensor array and fine-threaded screws [39] and loaded at the same normal load for an identical amount of time. At this point, photographs of the interface were taken at the central region of the frictional fault. We avoided the end regions of the interface (*i.e.*, *x* = 0−100 mm and *x* = 300−400 mm) to avoid the combination of shear and normal stress conditions that arise from the direct shear configuration [40].

**Figure 8:**
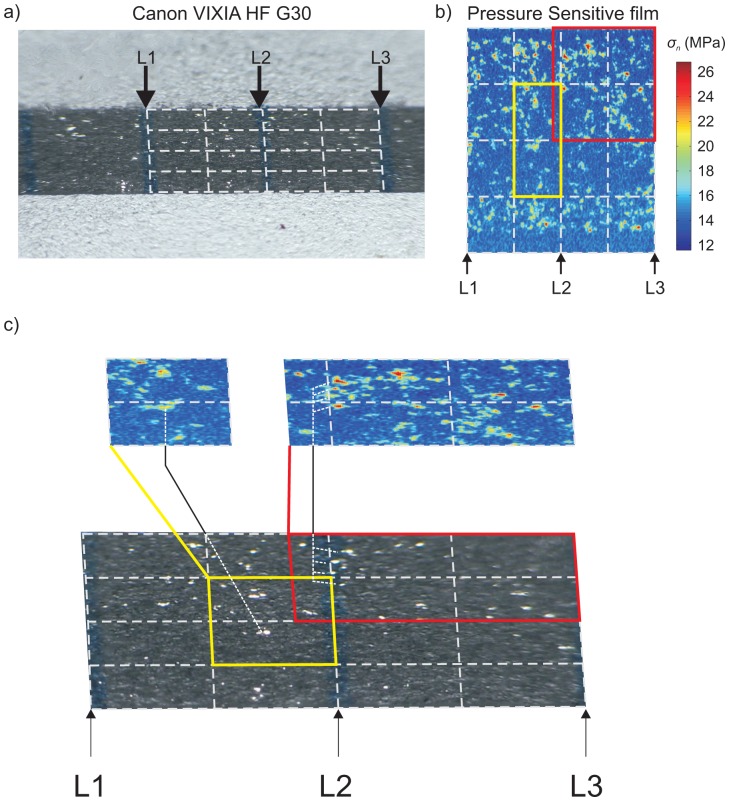
(**a**) A still frame obtained from the camcorder at one loading step. A grid was superimposed and followed the lines physically drawn on the interface. The lines L1, L2 and L3 were used for reference; (**b**) Results from the pressure-sensitive film (converted to stress) in the same location as in (a). Reference lines L1, L2 and L3 are shown. (**c**) The pressure-sensitive film was distorted using COREL DRAW to match the transformed coordinate system in the photograph (top). In the highlighted boxes, we see that the pressure measurements and the light transmitted occur at similar locations along the grid (connecting lines).

In Figure 8, we present a pre-processed photograph of the interface (Figure 8a) and the post-processed image of the same location using the pressure-sensitive film (Figure 8b) for the same applied normal load *F_n_*. The three lines, physically drawn along the interface (*i.e.*, L1, L2 and L3), were used to construct a grid (white dashed lines) along the interface that accounted for the orthoscopic distortions imposed from the camera, additional lens and PMMA sample. This grid was superimposed over the pressure film measurements in Figure 8b and referenced using lines L1, L2 and L3. Within the grid, two locations are highlighted in the pressure measurements (Figure 8b) using a yellow and red box. The pressure-sensitive film measurements were distorted using an image processing software (COREL DRAW X4), so that the distorted coordinate system matched those from the photographic images. In Figure 8c, locations where light is highly transmitted (in the photographic frame) are compared to locations where the normal stress appears to be higher (from the pressure film measurements). While not all regions exhibiting high levels of normal stress were concomitant, this was most likely due to experimental error and our inability to perfectly recreate the interface between when the film is extracted and when the photometric measurements are taken.

### 5.1. Calibration of Luminous Intensity to Normal Stress along Larger Asperities

**Figure 9:**
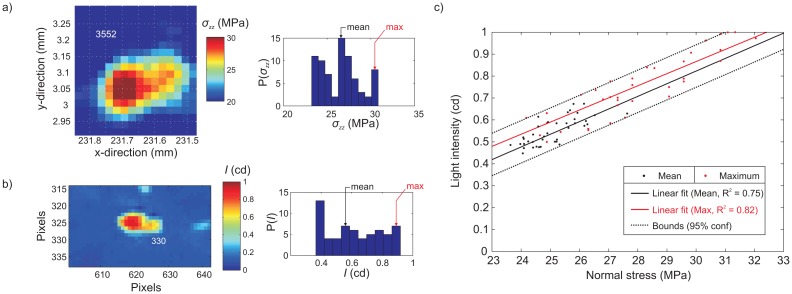
(**a**) Normal stress along an asperity measured using the pressure-sensitive film (left). On the right is the histogram of the stress for the same asperity; (**b**) Light intensity transmitted by the asperity in (a) measured using the video camera. The histogram for the light intensity is shown on the right. (**c**) The relation between normal stress and light intensity.

A total of 40 asperities that both transmitted light and had simultaneous film measurements of normal stress were analyzed for the same far-field normal load of *F_n_* ∼ 5.6 kN. These asperities were hand-picked, since the process of locating identical asperity distributions was not trivial. Figure 9a shows the normal stress distribution on a single asperity and the accompanying stress histogram. For the same asperity, we show the photometric measurement taken in Figure 9b with its accompanying histogram. For each asperity histogram, the maximum and mean normal stress and light intensity were recorded. Figure 8c shows the relation between normal stress and light intensity. Both the mean (black) and maximum (red) data points were plotted. Linear estimates of the maximum (*R*^2^ = 0.82) and mean (*R*^2^ = 0.75) relation between the normal stress and light intensity were plotted. We used the average of the two maximum and mean slopes and estimated the relation between light intensity and normal stress as follows: an increase of one MPa in normal stress caused the light intensity to increase 0.05943 ± 0.0012 cd between the normal stress range of 23–32 MPa with an error of ±1.45 MPa within a 95% confidence interval (dashed lines). In similar studies [13], the light transmitted was assumed only to occur on asperities that formed due to plastic contact, i.e., the stress levels were uniform over the entire asperity in accordance with the plastic contact limit *p_m_* [41]. We clearly see in each histogram (Figure 9a,b) that the range varies, a phenomena that can only be explained by elastic or elasto-plastic contact. The experimental error observed is most likely introduced when the pressure-sensitive film is extracted, and reconstituting identical asperity populations (in order to measure the light transmitted) was not fully achieved.

## 6. Application: Laboratory Earthquakes

**Figure 10:**
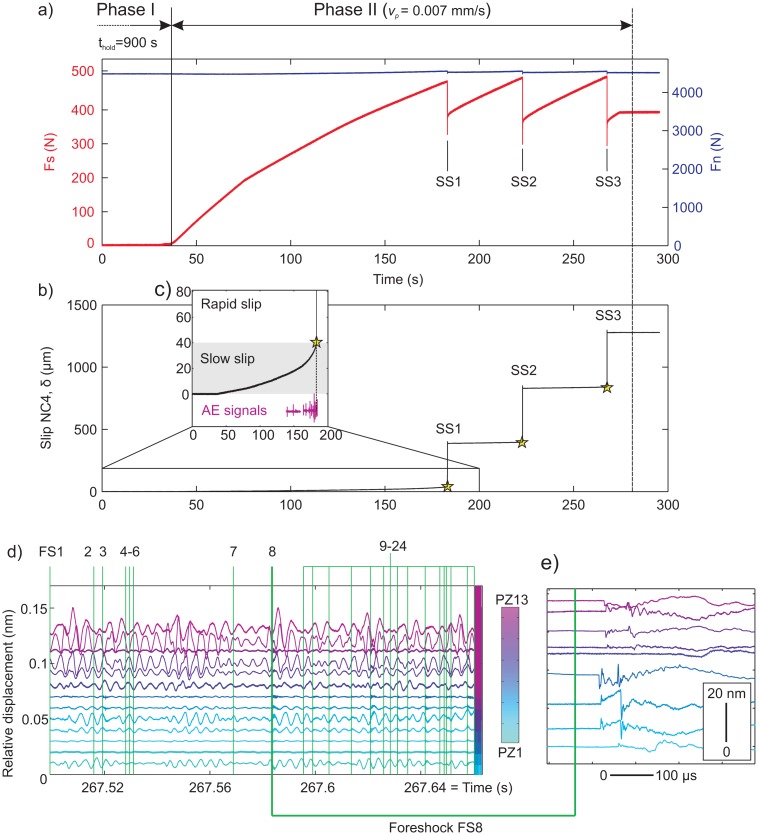
(**a**) Normal (blue) and shear (red) loads applied to the slider block during Phase II of the experiment. The rigid loading platen moved at *V_LP_* = 0.007 mm/s until a stick-slip event (SS). During an SS, the shear force dropped, while slip increased rapidly, as seen in (**b**). (**c**) Prior to an SS, we observed an accumulation of slow slip and localized, dynamic acoustic emission signals (AE) measured just prior to rapid slip. (**d**) The AE signals from piezoelectric sensors (PZ1–PZ13) are shown 209.7 ms before SS3. A total of 24 foreshocks were detected and located using the P wave components, such as that seen in (**e**).

Figure 10 shows a typical result for the direct shear experiment and the sensor measurements taken using the non-contact (NC) and acoustic emission (PZ) sensor array. Phase I of the direct shear experiment (see Figure 10a) consisted of loading the slider block against the base plate under a constant normal load *F_n_* = 4400 N for *t_hold_* = 900 s with no shear force *F_s_* applied (*V_LP_* = 0 mm/s). During Phase II, the normal load was maintained constant, and the rigid loading platen was driven at a constant velocity of *V_LP_* = 0.007 mm/s using the electro-mechanical shear actuator. Steady motion of the rigid loading platen was used to simulate the motion of tectonic plates, causing an accumulation of shear force *F_s_* along the interface. In Figure 10a, the normal force *F_n_* (blue) remained constant, while an observable increase in shear force F_s_ (red) occurs due to the motion of the rigid loading plate. As time continues, the bulk shear force *F_s_* increases until a stick-slip event (SS). Figure 10b shows slip displacements from one non-contact sensor (NC4) during three SSs (SS1, SS2 and SS3) that occurred during Phase II. Stick-slip events consisted of a sudden drop in shear force drops accompanied by rapid relative displacement across the interface. Figure 10c shows, in detail, the period leading up to a stick-slip, where slow slip built up prior to rapid sliding (instability). “Slow slip” was defined to be when local slip velocities are lower than *Υ_local_* > 10 mm/s (star symbol in Figure 10c), which was evaluated by differentiating the raw slip sensor data *a posteriori*. Superimposed on Figure 10c are the acoustic emission (magenta) measurements that occurred prior to SS1. These events are referred to as foreshocks (FS) and were present prior to the rapid sliding phase. Figure 10d shows the high-resolution AE signals (PZ1–PZ13) just prior to the SS3. Here, a total of 24 FS were detected prior to rapid sliding. Figure 10e shows a detailed view of FS8, emphasizing the pulse-like P and S wave arrivals intrinsic to all of these FS signals and indicative of the sudden release of stress waves from local dynamic failing of a single asperity [16,42].

### 6.1. Slow Slip Distribution and Foreshock Locations

**Figure 11:**
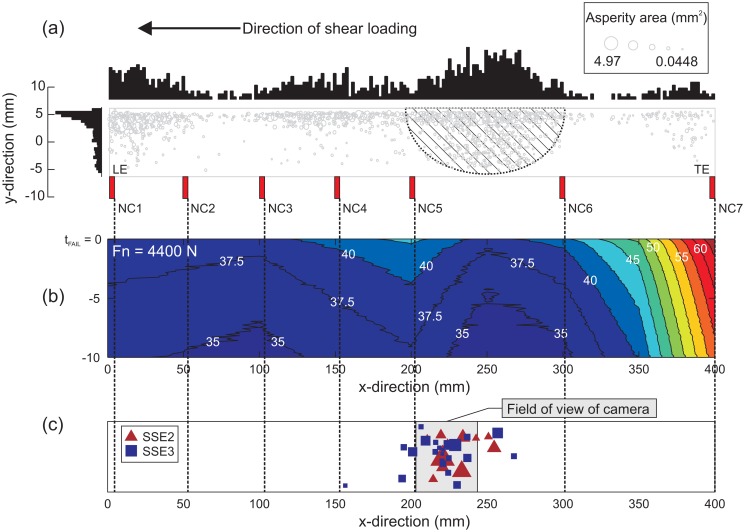
(**a**) Asperity locations are shown as circular patches on the interface with areas equivalent to that measured using the pressure-sensitive film; (**b**) slow slip measured along the fault using the slip non-contact sensors (NC1–NC7) over the last 10 s before rapid slip; (**c**) locations of foreshocks for SS2 (red triangles) and SS3 (blue squares). The sizes of the symbols are proportional to the magnitude of shaking from the event.

It has been found that the real distribution of asperities controls fault behavior for a dry friction fault [16]. Figure 11a shows estimated asperity locations measured using the pressure-sensitive film. While, in reality, asperities seem to form convoluted shapes [39,43], they are represented here as a circular patch with an equivalent area. Graphically, we represented a region of subdued slip by the hatched region shown in Figure 11a. Foreshocks occurred only in this hatched region, as seen in Figure 11c. Figure 11c shows the foreshock catalog for SS2 (red triangles) and SS3 (blue squares). The field of view (FOV) of the video camera is shown here in gray. The size of the symbols are proportional to the peak ground displacements (PGD) averaged over the closest three AE sensors used to locate the foreshock [16]. Figure 11b presents the accumulation of slow slip using the seven slip sensors prior to the first stick-slip event (SS1). Measurements on all seven slip sensors (NC1–NC7) were interpolated at 50-ms time intervals. The time associated with the onset of rapid slip according to our test, *i.e.*, *t_fail_* = 0 s, was the time that any sensors breached the slip velocity threshold of *Υ_local_* > 10 mm/s. The grid composed of slip values on all seven sensors (NC1–NC7) was then interpolated in time to provide visual insight into the slow-slip distributions. From Figure 11b, we see that slip began accumulating from the trailing edge (TE) region of the sample and propagated (relatively) slowly into the fault over the 10 s before tfail. This was likely caused by larger and denser distributions of asperities and the preferential loading conditions.

### 6.2. Optical Results in Relation to Localized Acoustic Events

The timing of the camera and the AE data acquisition was not synchronized *a priori* in this experiment. We rely solely on the frame-to-frame images and the changes in asperity transmitted luminous intensity between them. During the slow slip portion of the experiment, changes in the FOV occur slowly enough that images remain focused. During the transition and acceleration of shear rupture, the image suddenly becomes blurred. The first frame that was blurred was called the failure frame *F_tfail_*. The frames prior to these are referred to as *F_tfail_* − 1, *F_tfail_* − 2, *F_tfail_* − 3, *etc.*, that occurred at approximate times of *t_fail_* − 33.3 ms, *t_fail_* − 66.6 ms and *t_fail_* − 99.9 ms. We then examine the changes in light intensity in frames leading up to failure (*t_fail_*) during which foreshock sequences were observed (see Figure 11d). We consider only the SS2 foreshock (Figure 11c). The AE data showed that foreshock sequence SS2 occurred over a time span of 161.222 ms. Dividing by the frame rate of the camera (Δ*t_frame_*), we get 4.8 and 6.1, indicating that there was a minimum of four frames for the foreshock period.

**Figure 12:**
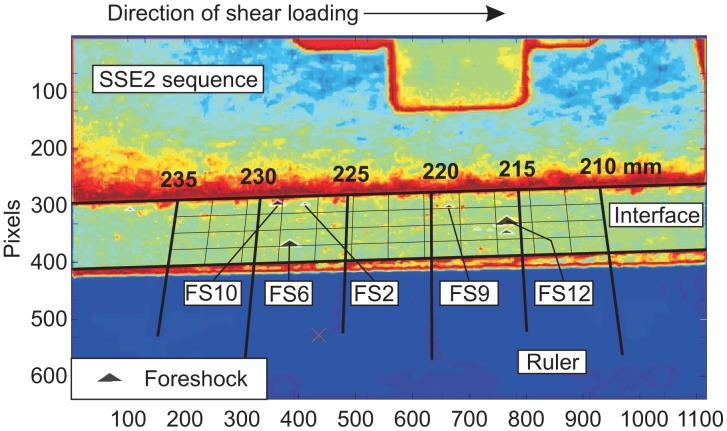
Images from the video camera at frame *F_tfail_* for SS2. A non-uniform grid was constructed using image-processing software, and foreshock locations were superimposed on each image. Shear loading was from left to right.

Figure 12 shows the failure frame, *F_tfail_* (for SS2), converted from a true-color image RGB to luminous intensity I in the same manner as described in Section 4 and inverted for clarity. The distorted grid accounted for orthoscopic distortions and was constructed along the interface using the same software as in Figure 8. In these images, the direction of shear loading was from left to right, and the location of the interface on the *x*-axis, referring to the coordinate system in Figure 11, was displayed above the grid. Only the foreshocks present in the field of view (FOV) are shown in Figure 11d. They are represented as black triangles, and their size is proportional to the peak ground displacement of each event [16]. The locations of foreshocks (FS), which aligned with asperities displaying higher luminous intensity, were analyzed in more detail.

**Figure 13:**
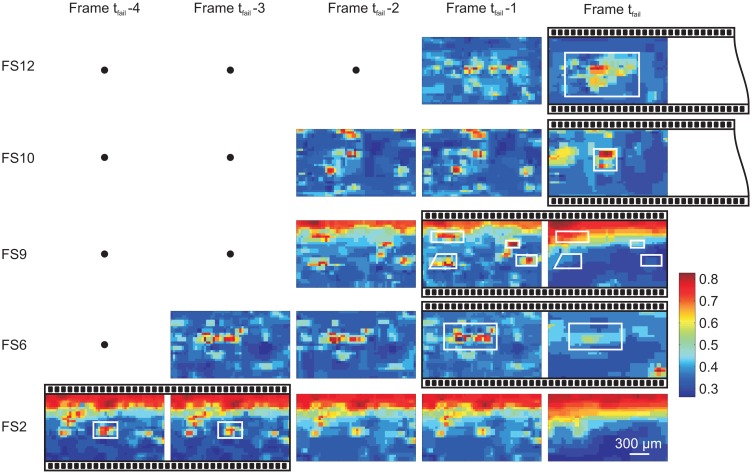
Final five frames prior to SS2 detailing the images taken in the location of each foreshock shown in Figure 12a. Changes in light intensity occurring between frames are highlighted by the bordered black boxes. Frame *F_tfail_* +1 was not shown, since it was blurred. White boxes give an estimate of the regions in which light intensity changed.

**Figure 14:**
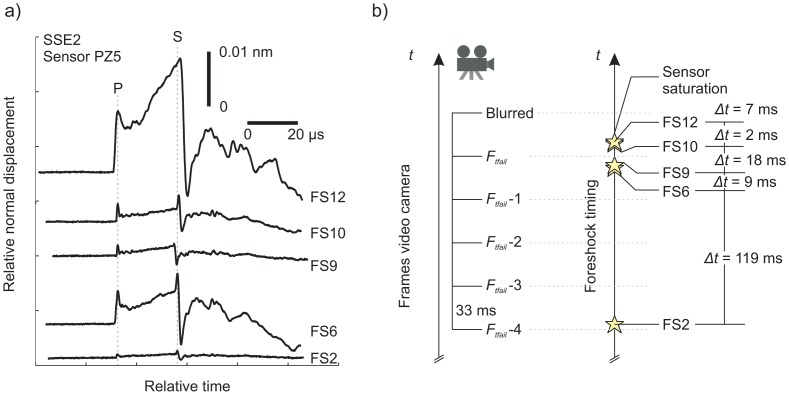
(**a**) AE signals from PZ5 for five foreshocks in the SS2 sequence, which were located in the field of view of the camera; (**b**) the left-hand side shows a schematic of the frame separation of the camera along time in the *y*-direction. Foreshocks shown in (a) are placed on the time axis on the right-hand side. Changes in light intensity due to foreshocks between frames allowed us to initialize the timing of the camera.

Now, we must examine the specific regions on the interface where the foreshocks were located, which is shown in Figure 13. For example, foreshocks FS6 and FS9 occur between frame *F_tfail_* and *F_tfail_* − 1 (according to Figure 13b), and there is a definitive change in light intensity near the location of the event. Now that an FS event from frame-to-frame (concomitant with the acoustic location in space) is located, we can move backwards and forwards in time, shifting the camera timing a maximum of Δ*t_frame_* − Δ(FS6−FS9), *i.e.*, 33 − (9) = 24 ms. This methodology is referred to as clapboard timing, used to appropriately align the timing of the video camera frames with the dynamic foreshocks measured acoustically. The clapboard timing approach gives a possible upper estimate on the true length scale of the region causing this dynamic signal (Figure 6a). Estimates of the x-direction length scale are shown using the white boxes in Figure 14 between the frame-to-frame changes. Note that more foreshocks spaced over a longer time interval would provide more accurate constraints on the clapboard timing of the video camera.

We focus on the capabilities (and limitations) of associating the sudden frame-to-frame changes in individual asperity luminous intensity with the location (and timing) of events detected using the acoustic array. In Figure 14, we look at the acoustic signals and attempt to reconcile the timing of foreshocks with respect to the images from the video camera. Figure 14a shows the AE signals from sensor PZ5 for five foreshocks (FS2, FS6, FS9, FS10 and FS12) within the cameras field of view during SS2. These foreshocks have also been marked spatially in Figure 12 (top). In Figure 14b, the right-hand side shows the timing of foreshocks determined using the acoustic data, and the time between each foreshock was shown (Δ*t*). To the left are the images taken from the camera from frame *F_tfail_* to *F_tfail_* 4, and the spacing between each frame was Δ*t_frame_* (33 ms). Foreshock signals have been aligned about the peak P-wave arrival and in ascending order of occurrence along the increasing y-axis. We assumed that these signals are created from the sudden failure of asperities along the interface [17,42]. If this is the case, we expect to see sudden frame-to-frame changes in the luminous intensity passing through the asperity after the foreshock due to either the destruction (or partial destruction) of the asperity.

## 7. Conclusions

We presented three novel methodologies to better understand random contact processes occurring when an interface is created between two rough surfaces. The ability to develop a better understanding depends entirely on the sensors employed and the user's ability to properly interpret (*i.e.*, calibrate) the signals produced. Friction caused between two contacting surfaces is a problem that is misunderstood and widely studied in both engineering and scientific disciplines. In this study, we used a simple contact problem and determined that the pressure-sensitive film is a valid sensor to measure the normal stress distribution over a relatively small spatial resolution (micron length scale). Once validated, the film was used to estimate the relation between photometric light transmitted through contacting asperities and its dependence on the local normal stress. We found that increased normal stress caused increased transmission of luminous intensity for a known normal stress range. Photometry of the asperities along the interface becomes a valid tool for investigating dynamic stress changes in more intricate tests. The calibration of the pressure-sensitive film and photometric light measurements was done under simple normal loading.

We utilized both the non-contact sensor and the acoustic arrays during a direct shear experiment. Before the test, the pressure-sensitive film was used to characterize the interface, and during the test, the video camera took asperity photometric measurements from a specific “region of interest”. Shear stress accumulated along the fault until failure, *i.e.*, a stick-slip event, where slow slip accrued non-uniformly along the fault. Within the “region of interest” (focused on by the camera), we observed decreased amounts of slow slip and foreshock events measured acoustically. The camera, focused on this region beforehand, capturing sudden changes in asperity luminosity occurring from frame-to-frame in the video prior to failure. These changes were concomitant, in both space and time, with the localized foreshocks measured acoustically. Using the acousto-optical method presented here, we display our ability to measure the sudden changes in light intensity caused from the sudden failure (or partial failure) of asperities. This is the first time that an acoustic emission event has been absolutely identified that was visually concomitant with respect to a physical event having known dynamics on a frictional fault.

The fact that the prescale pressure-sensitive film has been calibrated independently may move experimenters to apply it to their projects. While possibly of less utility, the optical method can be an important tool to calibrate the contact surface being worked with. Our results show the film's ability to estimate roughness characteristics along surfaces. We believe that the film can be employed to assess the health of a structural component that may be susceptible to wear or attrition. It is especially useful for the fitting and testing of flanges on pipelines and other precision jointings. For SHM, this allows quantification of corrosion. The film is ideal for quantifying track conditions, important for high-speed rail. Equipment that requires very smooth surfaces, such as turbines and ship props, experiences pitting caused by cavitation of the fluid near its surface that can be captured by the film. We believe that the methods presented in this paper have great possibilities for many researchers.
